# Supplementary material for: Predicting bacterial phenotypic traits through improved machine learning using high-quality, curated datasets
Source: Commun Biol. 2025 Jun 7;8:897. doi: 10.1038/s42003-025-08313-3 (PMC12145430; doi:10.1038/s42003-025-08313-3)
Supplement: Supplementary file 2 — Supplementary information [file 42003_2025_8313_MOESM2_ESM.pdf]

Supplementary Information for

Predicting bacterial phenotypic traits through improved machine learning using high-quality, curated datasets

Julia Koblitz *et al.*

*Corresponding author. Email: julia.koblitz@dsmz.de

This PDF file includes:

Figs. S1 to S5
Tables S1 to S1

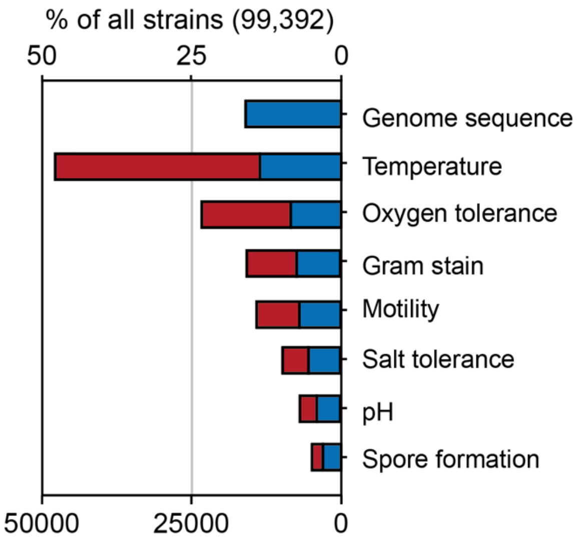

Figure S1: Availability of genome sequences and most common types of phenotypic information for all bacterial strains covered by the Bac*Dive* database. In contrast to figure 1 of the main manuscript, the strains are not limited to type strains. Records are colored blue if a genome is present and red if not.

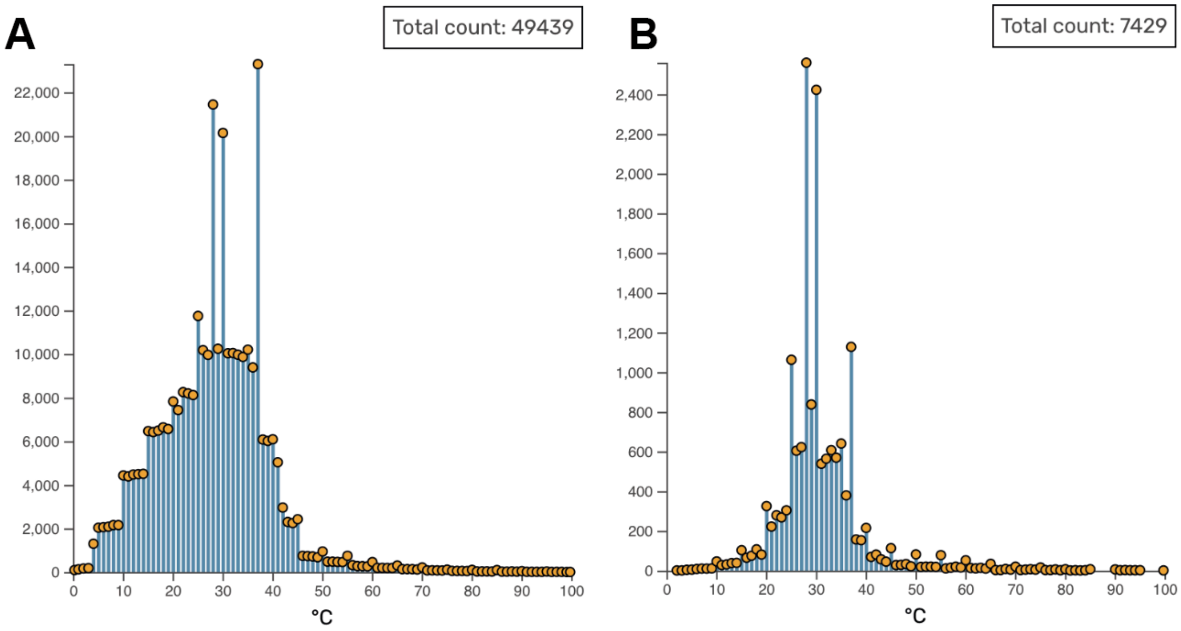

Figure S2: Overview on temperature data from Bac*Dive*. Screenshots were taken on the statistics dashboard of the BacDive database (https://bacdive.de/dashboard) on May 23, 2025. **(A)** All growth temperature datasets. **(B)** Growth temperature datasets limited to optimum temperature.

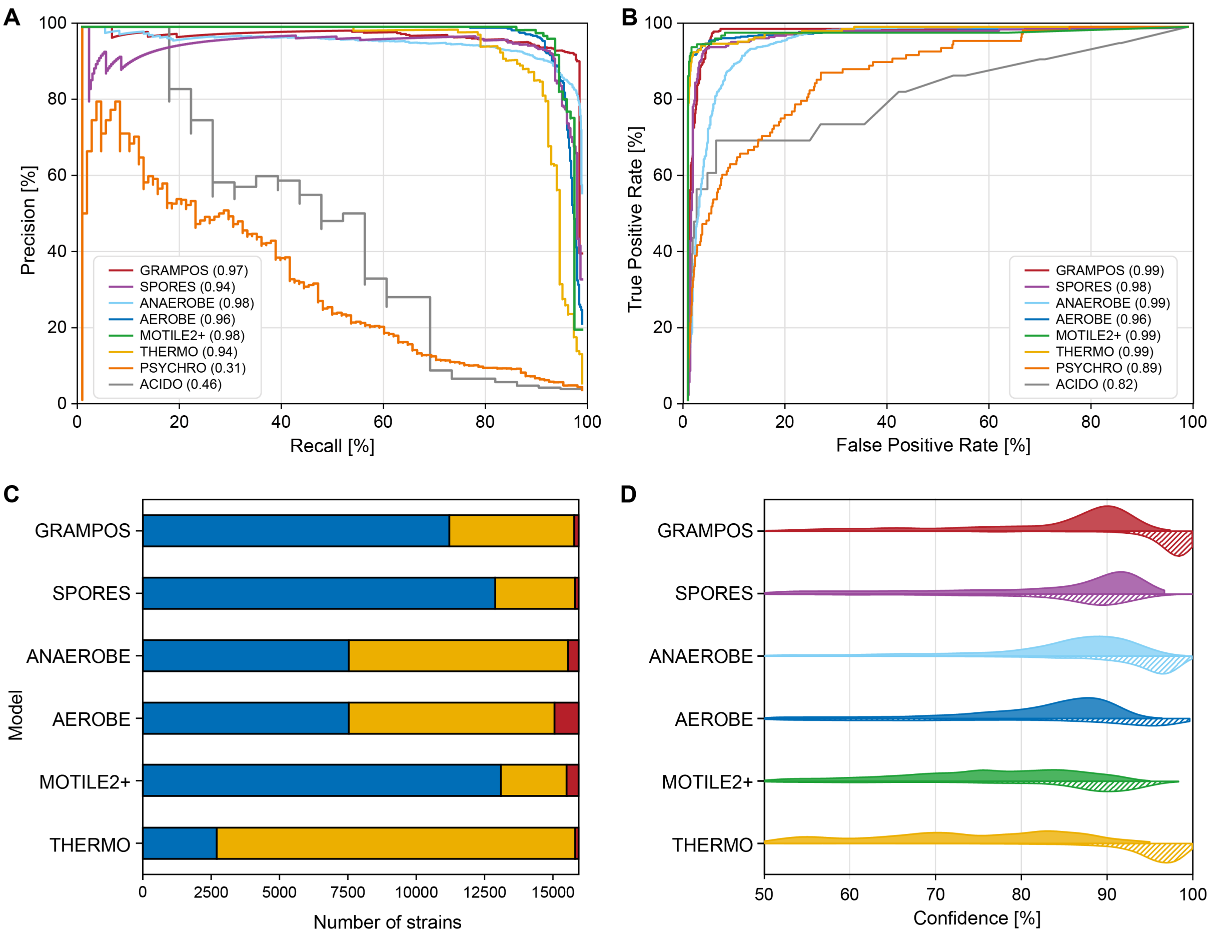

Figure S3: Model performance metrics and improvement of the Bac*Dive* database with the models from this study. (A) Precision/recall curves indicate model performance. The legend also contains the calculated AUPR. **(B)** The ROC curve is an often-used measure but less reliable for unbalanced datasets, such as ACIDO and PSYCHRO. The legend also contains the calculated AUC. **(C)** The number of strains to which prediction data has been added. Blue: new data where the strain did not have information on the trait before, green: consistent data where the predicted label is consistent with the manually curated information, red: contradicting data where the prediction contradicts the curated data. **(D)** Violin plots showing the confidence distributions for each dataset in (C). Hatched: negative predictions, filled: positive predictions.

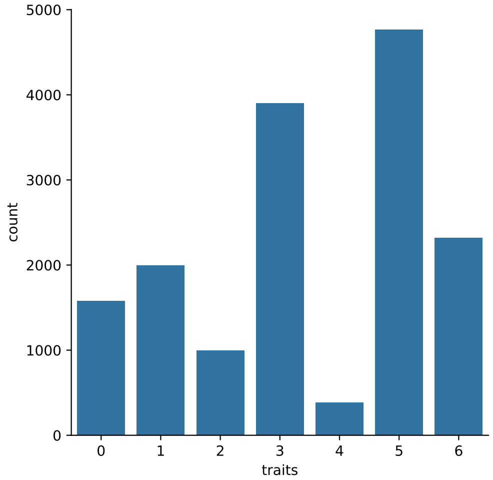

Figure S4: Number of new traits that were added to strains in Bac*Dive*. 0 means no new data points were added to a strain, 6 means the strain had none of the phenotypic data before.

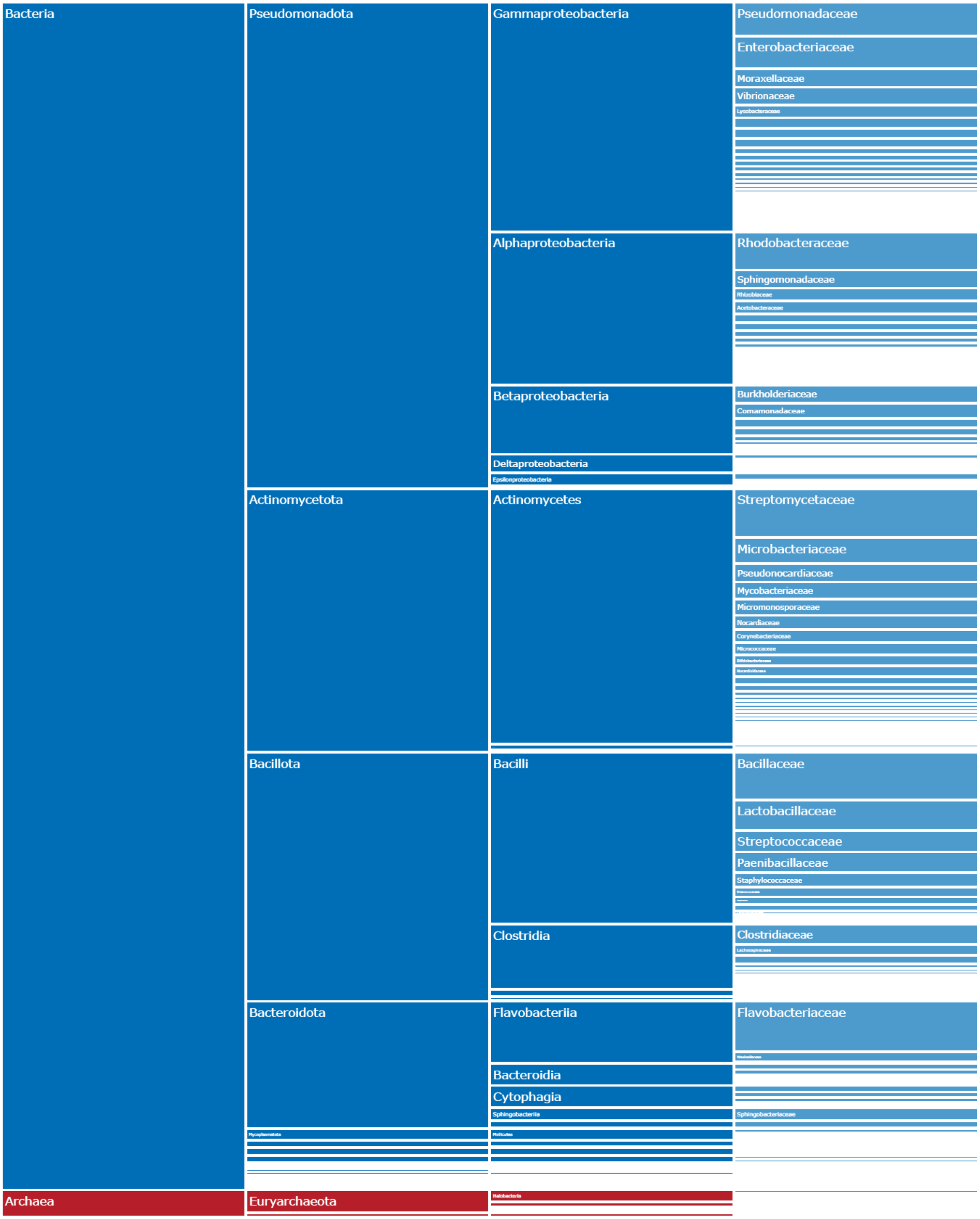

Figure S5: Taxonomic classification of all strains used in the training and test dataset. Taxonomic names according to LPSN.

Table S1: Feature importances of the MOTILE model.

Pfam	Description	Importance
PF00771	FHIPEP family	0.024
PF02154	Flagellar motor switch protein FliM	0.023
PF06429	Flagellar basal body rod FlgEFG protein C-terminal	0.022
PF01514	Secretory protein of YscJ/FliF family	0.021
PF00669	Bacterial flagellin N-terminal helical region	0.020
PF14841	FliG middle domain	0.020
PF02049	Flagellar hook-basal body complex protein FliE	0.019
PF01311	Bacterial export proteins, family 1	0.018
PF00813	FliP family	0.015
PF01313	Bacterial export proteins, family 3	0.014
PF07195	Flagellar hook-associated protein 2 C-terminus	0.013
PF01052	Type III flagellar switch regulator (C-ring) FliN C-term	0.013
PF00015	Methyl-accepting chemotaxis protein (MCP) signalling domain	0.013
PF14842	FliG N-terminal domain	0.013
PF01312	FlhB HrpN YscU SpaS Family	0.012
PF01706	FliG C-terminal domain	0.012
PF01584	CheW-like domain	0.011
PF00700	Bacterial flagellin C-terminal helical region	0.011
PF18269	T3SS EscN ATPase C-terminal domain	0.009
PF08345	Flagellar M-ring protein C-terminal	0.009
PF03963	Flagellar hook capping protein - N-terminal region	0.008
PF00033	Cytochrome b/b6/petB	0.008
PF02119	Flagellar P-ring protein	0.007
PF06798	PrkA serine protein kinase C-terminal domain	0.007
PF02561	Flagellar protein FliS	0.007

Table S2: Mobility test of selected strains with 21 flagellum Pfams that were reported as non-motile in the literature.

Strain	Bac*Dive*-ID	Confidence (MOTILE_2)	Motility	Reference
Heyndrickxia oleronia DSM 9356	1102	84.6	++	(*32*)
Halobacillus kuroshimensis DSM 18393	1349	84.7	+	(*33*)
Salipaludibacillus neizhouensis DSM 19794	1293	83.0	(+)	(*34*, *35*)
Terribacillus halophilus DSM 21620	1518	81.9	(+)	(*36*)
Priestia endophytica DSM 13796	1203	78.6	-	(*37*)
